# Supplementary material for: Classification of Visual Cortex Plasticity Phenotypes following Treatment for Amblyopia
Source: Neural Plast. 2019 Sep 3;2019:2564018. doi: 10.1155/2019/2564018 (PMC6746165; doi:10.1155/2019/2564018)
Supplement: Supplementary Materials — Supplemental Tables 2-1 and 2-2 expand on the descriptive statistics for the rearing conditions outlined in Table 2 in the main text. Supplemental Figure 7 describes the method used to determine the number of clusters identified after tSNE analysis. The remaining tables in the supplemental material contain supporting statistics that accompany Figures 3–6, 8, 10, and 11. [file 2564018.f1.pdf]

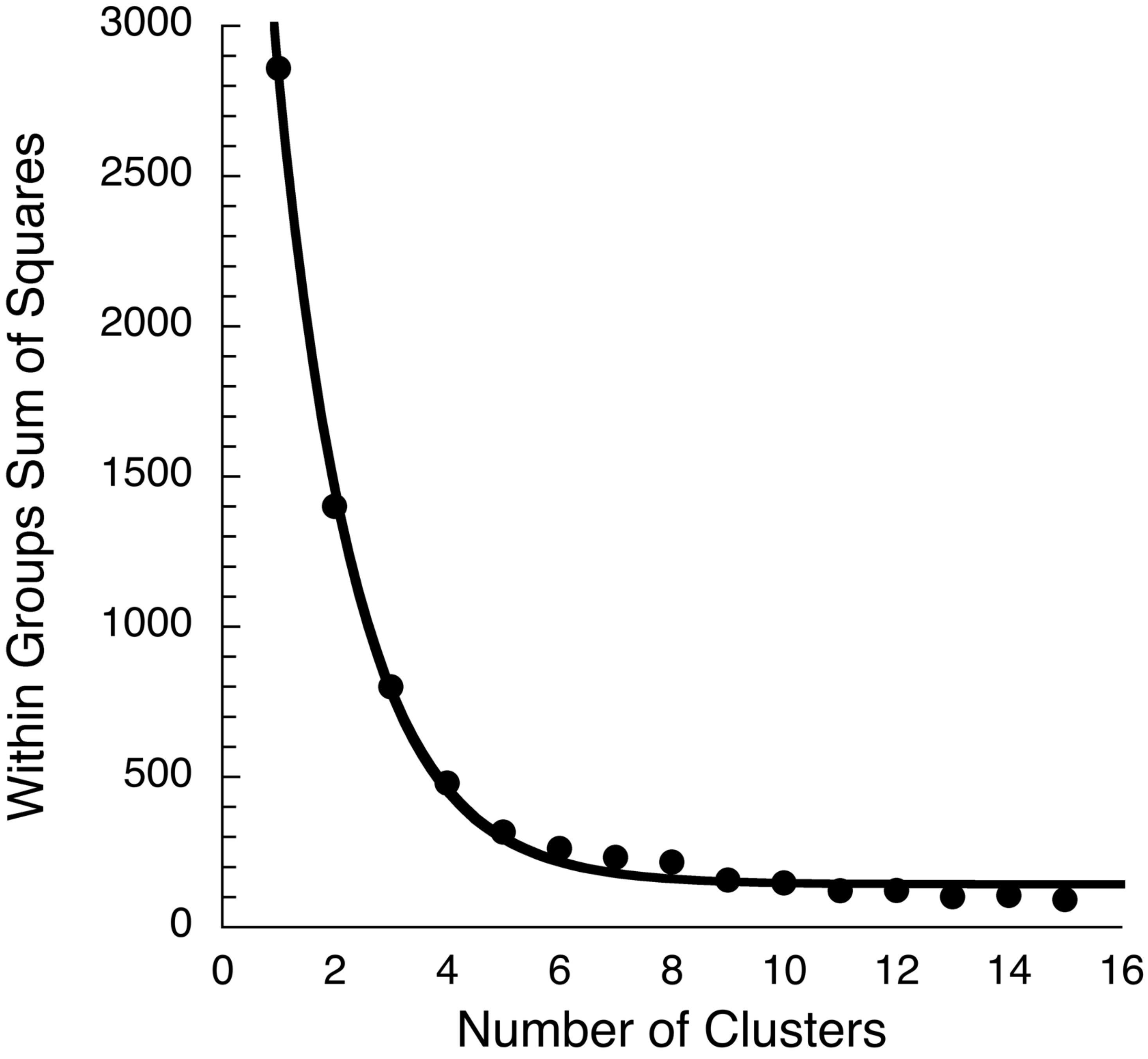

Figure 7-1 Within group sum of squares with variable cluster sizes. Scatterplot of the within groups sum of squares was measured across a range of clusters between 2 and 15. An exponential decay fit was applied to the data, and 4τ was taken as the point at which changes in cluster number had little effect on the within groups sum of squares. The optimal number of clusters was identified as 6 (k=6). This value was used to assign the k-means clusters on tSNE reduced data (Figure 7a).

Weeks BV	Region	GluN1	GluN2A	GluN2B	$GABA_A\alpha1$	$GABA_A\alpha3$	GluA2	Synapsin
2	C	4	4	4	4	4	4	4
	P	16	16	16	16	16	16	16
	M	4	4	4	4	4	4	4
3	C	4	4	4	4	4	4	4
	P	16	16	16	16	16	16	16
	M	4	4	4	4	4	4	4
4	C	4	4	4	4	4	4	4
	P	16	16	16	16	16	16	16
	M	4	4	4	4	4	4	4
5	C	4	4	4	4	4	4	4
	P	16	16	16	15	16	16	16
	M	4	4	4	4	4	4	4
6	C	4	4	4	4	4	4	4
	P	16	16	16	16	16	16	16
	M	4	4	4	4	4	4	4
8	C	6	6	6	6	6	6	6
	P	22	19	22	22	22	22	22
	M	2	2	2	2	2	2	2
12	C	4	4	4	4	4	4	4
	P	16	16	16	16	16	13	16
	M	4	4	4	4	4	4	4
16	C	4	4	4	4	4	4	4
	P	16	16	16	16	16	16	16
	M	4	4	4	4	4	4	4
32	C	4	4	4	4	4	4	4
	P	16	16	16	16	16	16	16
	M	4	4	4	4	4	4	4
SUM		222	219	222	221	222	219	222

Table 2-1. The number of Western blot measurements for each cortical region in Normal animals. Rows summarize the number of runs from the Central (C), Peripheral (P), and Monocular (M) regions of V1 within each age of animal studied. The columns list each of the 7 proteins analyzed using Western blotting. Column sums detail the number of runs across ages and cortical areas.

Weeks MD	Region	GluN1	GluN2A	GluN2B	GABA$_A$α1	GABA$_A$α3	GluA2	Synapsin
4	C	4	4	4	4	4	4	4
	P	16	16	16	16	16	11	16
	M	4	4	4	4	4	2	4
5	C	6	6	6	6	6	6	4
	P	18	18	18	18	18	18	11
	M	4	4	4	4	4	4	4
6	C	8	8	8	8	8	8	8
	P	32	32	32	32	32	32	32
	M	6	6	6	6	6	6	6
9	C	8	8	8	8	8	8	8
	P	36	36	34	34	36	36	31
	M	8	8	8	6	8	8	6
32	C	4	4	4	4	4	4	4
	P	16	16	16	16	16	16	16
	M	2	2	2	2	2	2	2
SUM		172	172	170	168	172	165	156

Table 2-2. The number of Western blot measurements for each cortical region in MD animals. Rows summarize the number of runs from the Central (C), Peripheral (P), and Monocular (M) regions of V1 within each age of animal studied. The columns list each of the 7 proteins analyzed using Western blotting. Column sums detail the number of runs across ages and cortical areas.

		Pearson's R							Bonferroni Corrected p-value						
		GluN1	GluN2A	GluN2B	GABA$_A$α1	GABA$_A$α3	GluA2	Synapsin	GluN1	GluN2A	GluN2B	GABA$_A$α1	GABA$_A$α3	GluA2	Synapsin
	**GluN1**	1.0000	0.1244	0.7605	**0.8623**	0.6852	0.6861	**0.8459**		14.7036	0.0857	0.0065	0.2927	0.2887	0.0110
	**GluN2A**	0.1244	1.0000	0.4563	0.0831	0.2667	0.4129	0.1248	14.7036		2.8551	16.7446	8.4450	3.8260	14.6803
	**GluN2B**	0.7605	0.4563	1.0000	0.7056	0.6757	0.7619	0.6297	0.0857	2.8551		0.2176	0.3334	0.0836	0.5924
5wk Normal	**GABA$_A$α1**	**0.8623**	0.0831	0.7056	1.0000	0.7158	0.6524	0.6873	0.0065	16.7446	0.2176		0.1857	0.4512	0.2839
	**GABA$_A$α3**	0.6852	0.2667	0.6757	0.7158	1.0000	**0.9535**	0.7488	0.2927	8.4450	0.3334	0.1857		0.0000	0.1065
	**GluA2**	0.6861	0.4129	0.7619	0.6524	**0.9535**	1.0000	0.7454	0.2887	3.8260	0.0836	0.4512	0.0000		0.1133
	**Synapsin**	**0.8459**	0.1248	0.6297	0.6873	0.7488	0.7454	1.0000	0.0110	14.6803	0.5924	0.2839	0.1065	0.1133	
	**GluN1**	1.0000	**0.4100**	**0.6353**	-0.7834	-0.5047	0.0634	-0.7529		0.0018	0.0002	3.1854	4.6850	0.0736	16.8673
	**GluN2A**	**0.4100**	1.0000	**0.2805**	0.1070	-0.0803	**0.4764**	-0.4470	0.0018		0.0006	15.3799	0.0675	0.0000	5.4053
	**GluN2B**	**0.6353**	**0.2805**	1.0000	-0.3743	-0.5441	**0.2998**	-0.2972	0.0002	0.0006		16.0847	2.4495	0.0017	8.5682
5wk MD	**GABA$_A$α1**	-0.7834	0.1070	-0.3743	1.0000	0.5027	0.4173	0.6579	3.1854	15.3799	16.0847		4.9244	9.0319	0.1331
	**GABA$_A$α3**	-0.5047	-0.0803	-0.5441	0.5027	1.0000	-0.0448	0.2924	4.6850	0.0675	2.4495	4.9244		0.0689	4.0109
	**GluA2**	0.0634	**0.4764**	**0.2998**	0.4173	-0.0448	1.0000	0.2912	0.0736	0.0000	0.0017	9.0319	0.0689		0.6893
	**Synapsin**	-0.7529	-0.4470	-0.2972	0.6579	0.2924	0.2912	1.0000	16.8673	5.4053	8.5682	0.1331	4.0109	0.6893	
	**GluN1**	1.0000	0.6894	0.4321	0.3385	0.0863	0.1573	0.6450		0.1339	2.5805	4.9665	16.1523	12.4138	0.2679
	**GluN2A**	0.6894	1.0000	0.6420	-0.0955	-0.0075	0.4625	**0.7514**	0.1339		0.2794	15.6551	20.5761	2.0130	0.0408
	**GluN2B**	0.4321	0.6420	1.0000	0.1985	0.3214	0.0836	0.3557	2.5805	0.2794		10.4227	5.5118	16.3023	4.4506
RO	**GABA$_A$α1**	0.3385	-0.0955	0.1985	1.0000	0.5621	-0.0245	0.3207	4.9665	15.6551	10.4227		0.7652	19.6111	5.5355
	**GABA$_A$α3**	0.0863	-0.0075	0.3214	0.5621	1.0000	-0.0713	0.2312	16.1523	20.5761	5.5118	0.7652		16.9808	8.9548
	**GluA2**	0.1573	0.4625	0.0836	-0.0245	-0.0713	1.0000	0.4624	12.4138	2.0130	16.3023	19.6111	16.9808		2.0148
	**Synapsin**	0.6450	**0.7514**	0.3557	0.3207	0.2312	0.4624	1.0000	0.2679	0.0408	4.4506	5.5355	8.9548	2.0148	
	**GluN1**	1.0000	**0.7646**	**0.7614**	0.5617	0.5436	**0.7744**	-0.2661		0.0304	0.0327	0.7683	0.9345	0.0241	7.5155
	**GluN2A**	**0.7646**	1.0000	0.5647	0.2690	0.1798	0.4188	-0.0405	0.0304		0.7433	7.3989	11.3082	2.8587	18.7055
	**GluN2B**	**0.7614**	0.5647	1.0000	0.5020	0.6689	**0.8751**	-0.3286	0.0327	0.7433		1.4143	0.1868	0.0009	5.2770
BD	**GABA$_A$α1**	0.5617	0.2690	0.5020	1.0000	**0.7619**	0.5675	-0.4707	0.7683	7.3989	1.4143		0.0324	0.7197	1.8771
	**GABA$_A$α3**	0.5436	0.1798	0.6689	**0.7619**	1.0000	**0.7979**	-0.4945	0.9345	11.3082	0.1868	0.0324		0.0131	1.5173
	**GluA2**	**0.7744**	0.4188	**0.8751**	0.5675	**0.7979**	1.0000	-0.4630	0.0241	2.8587	0.0009	0.7197	0.0131		2.0049
	**Synapsin**	-0.2661	-0.0405	-0.3286	-0.4707	-0.4945	-0.4630	1.0000	7.5155	18.7055	5.2770	1.8771	1.5173	2.0049	
	**GluN1**	1.0000	0.3855	0.6080	0.2625	0.3749	0.3297	0.3648		4.5343	0.7552	8.6073	4.8274	6.2014	5.1157
	**GluN2A**	0.3855	1.0000	0.5765	0.4831	0.5649	0.5557	-0.3930	4.5343		1.0443	2.3441	1.1682	1.2743	4.3313
	**GluN2B**	0.6080	0.5765	1.0000	0.5026	0.6824	0.6945	0.1850	0.7552	1.0443		2.0127	0.3043	0.2562	11.8611
1hr BV	**GABA$_A$α1**	0.2625	0.4831	0.5026	1.0000	0.7756	0.6488	-0.3023	8.6073	2.3441	2.0127		0.0636	0.4717	7.1321
	**GABA$_A$α3**	0.3749	0.5649	0.6824	0.7756	1.0000	0.7779	-0.2078	4.8274	1.1682	0.3043	0.0636		0.0607	10.8577
	**GluA2**	0.3297	0.5557	0.6945	0.6488	0.7779	1.0000	-0.2947	6.2014	1.2743	0.2562	0.4717	0.0607		7.4014
	**Synapsin**	0.3648	-0.3930	0.1850	-0.3023	-0.2078	-0.2947	1.0000	5.1157	4.3313	11.8611	7.1321	10.8577	7.4014	
	**GluN1**	1.0000	0.5988	0.3296	**0.7883**	0.4455	0.7283	-0.7436		0.8326	6.2047	0.0487	3.0797	0.1519	0.1170
	**GluN2A**	0.5988	1.0000	0.3423	0.3607	0.2277	0.6980	-0.7610	0.8326		5.7978	5.2382	10.0069	0.2436	0.0849
	**GluN2B**	0.3296	0.3423	1.0000	-0.0006	0.3319	0.1969	-0.0005	6.2047	5.7978		20.9706	6.1304	11.3330	20.9738
6hr BV	**GABA$_A$α1**	**0.7883**	0.3607	-0.0006	1.0000	0.4846	0.6657	-0.7018	0.0487	5.2382	20.9706		2.3178	0.3806	0.2301
	**GABA$_A$α3**	0.4455	0.2277	0.3319	0.4846	1.0000	0.4798	-0.1380	3.0797	10.0069	6.1304	2.3178		2.4036	14.0483
	**GluA2**	0.7283	0.6980	0.1969	0.6657	0.4798	1.0000	**-0.8150**	0.1519	0.2436	11.3330	0.3806	2.4036		0.0260
	**Synapsin**	-0.7436	-0.7610	-0.0005	-0.7018	-0.1380	**-0.8150**	1.0000	0.1170	0.0849	20.9738	0.2301	14.0483	0.0260	
	**GluN1**	1.0000	0.6855	0.6145	0.2300	**0.9282**	0.6501	-0.4983		0.2911	0.7035	9.9149	0.0003	0.4639	2.0834
	**GluN2A**	0.6855	1.0000	0.0939	-0.1271	0.5680	0.2423	-0.3108	0.2911		16.2063	14.5689	1.1342	9.4078	6.8364
	**GluN2B**	0.6145	0.0939	1.0000	0.3267	0.4719	0.4137	-0.2796	0.7035	16.2063		6.2991	2.5503	3.8076	7.9555
1d BV	**GABA$_A$α1**	0.2300	-0.1271	0.3267	1.0000	0.2823	0.6785	-0.4166	9.9149	14.5689	6.2991		7.8546	0.3209	3.7368
	**GABA$_A$α3**	**0.9282**	0.5680	0.4719	0.2823	1.0000	0.7387	-0.4850	0.0003	1.1342	2.5503	7.8546		0.1275	2.3104
	**GluA2**	0.6501	0.2423	0.4137	0.6785	0.7387	1.0000	-0.2997	0.4639	9.4078	3.8076	0.3209	0.1275		7.2214
	**Synapsin**	-0.4983	-0.3108	-0.2796	-0.4166	-0.4850	-0.2997	1.0000	2.0834	6.8364	7.9555	3.7368	2.3104	7.2214	
	**GluN1**	1.0000	0.5828	0.7563	0.4025	0.5404	0.5221	-0.3177		0.9818	0.0929	4.0868	1.4634	1.7143	6.6013
	**GluN2A**	0.5828	1.0000	0.5211	0.3540	0.6509	0.1215	0.1846	0.9818		1.7288	5.4374	0.4596	14.8432	11.8825
	**GluN2B**	0.7563	0.5211	1.0000	0.5761	0.4723	0.2560	-0.3347	0.0929	1.7288		1.0490	2.5424	8.8604	6.0401
2d BV	**GABA$_A$α1**	0.4025	0.3540	0.5761	1.0000	-0.1227	-0.1081	-0.2650	4.0868	5.4374	1.0490		14.7830	15.4998	8.5092
	**GABA$_A$α3**	0.5404	0.6509	0.4723	-0.1227	1.0000	0.3566	0.0017	1.4634	0.4596	2.5424	14.7830		5.3577	20.9110
	**GluA2**	0.5221	0.1215	0.2560	-0.1081	0.3566	1.0000	-0.5480	1.7143	14.8432	8.8604	15.4998	5.3577		1.3666
	**Synapsin**	-0.3177	0.1846	-0.3347	-0.2650	0.0017	-0.5480	1.0000	6.6013	11.8825	6.0401	8.5092	20.9110	1.3666	
	**GluN1**	1.0000	0.7597	0.7097	-0.2995	0.2312	**0.8860**	0.2433		0.2266	0.4518	8.4096	10.9292	0.0135	10.4634
	**GluN2A**	0.7597	1.0000	**0.9083**	-0.1464	0.2666	0.7004	0.3736	0.2266		0.0058	14.4161	9.5874	0.5058	6.0387
	**GluN2B**	0.7097	**0.9083**	1.0000	-0.0472	0.2951	0.7536	0.5582	0.4518	0.0058		18.8348	8.5644	0.2484	1.9639
4d BV	**GABA$_A$α1**	-0.2995	-0.1464	-0.0472	1.0000	0.4077	-0.1449	0.4367	8.4096	14.4161	18.8348		5.0863	14.4833	4.3467
	**GABA$_A$α3**	0.2312	0.2666	0.2951	0.4077	1.0000	0.0029	0.5863	10.9292	9.5874	8.5644	5.0863		20.8653	1.5720
	**GluA2**	**0.8860**	0.7004	0.7536	-0.1449	0.0029	1.0000	0.3818	0.0135	0.5058	0.2484	14.4833	20.8653		5.8017
	**Synapsin**	0.2433	0.3736	0.5582	0.4367	0.5863	0.3818	1.0000	10.4634	6.0387	1.9639	4.3467	1.5720	5.8017	

Table 3-1. Pearson's R values in each treatment condition comparing the strength of association between each protein. The correlation between each protein within a treatment condition was measured (left), and the observed R values are presented in a matrix. This matrix was reordered in Figure 3 to position high R values nearest one another. The Bonferroni corrected p-values (right) were used to identify the most significant correlations between proteins. P-values <0.05 are colored red to simplify identification of significant correlations.

	All Protein Sum	GlutR Sum	$GABA_AR$ Sum	GluN2B:GluN2A Index	$GABA_A\alpha3:GABA_A\alpha1$ Index	$GABA_A\alpha1$:GluN2A Index	GluN2B:GluA2 Index	GluN2A:GluA2 Index	GlutR Sum:$GABA_AR$ Sum Index	GluA2:GluN1 Index
Dim.1	0.0000	0.0000	0.0000	1.0000	0.0930	0.0000	1.0000	1.0000	1.0000	1.0000
Dim.2	0.7541	0.2225	0.0000	0.0000	0.0000	0.0000	0.0134	0.1211	1.0000	1.0000
Dim.3	1.0000	0.0000	1.0000	0.0000	0.0336	1.0000	0.0000	0.0000	1.0000	1.0000

Table 4-1. Pearson's R correlations between newly identified plasticity features and PCA dimensions. The correlation between the PCA scores across all animals, and the first 3 PCA dimensions are presented. P-values of correlations that were significantly correlated after Bonferroni correction are coloured red.

		Central					Peripheral					Monocular				
		Vs 5wk Normal		vs. 5wk MD		Curve Fit to BV Data	Vs 5wk Normal		vs. 5wk MD		Curve Fit to BV Data	Vs 5wk Normal		vs. 5wk MD		Curve Fit to BV Data
		Significance	p-value	Significance	p-value		Significance	p-value	Significance	p-value		Significance	p-value	Significance	p-value	
**Total Protein Sum**	**MD**	n.s.	0.3039				n.s.	0.1149				***	0.0000			
	**RO**	n.s.	0.0963	n.s.	0.1663		***	0.0000	***	0.0000		n.s.	0.4685	**	0.0089	
	**BD**	n.s.	0.1937	n.s.	0.2379		n.s.	0.2561	n.s.	0.1308		**	0.0077	n.s.	0.4364	
	**1hr BV**	***	0.0006	**	0.0024		***	0.0000	***	0.0000		***	0.0000	***	0.0000	
	**6hr BV**	n.s.	0.0825	n.s.	0.1652		n.s.	0.1939	n.s.	0.3297		**	0.0048	n.s.	0.3811	
	**1d BV**	**	0.0062	*	0.0151		***	0.0000	***	0.0000		n.s.	0.0664	***	0.0000	
	**2d BV**	n.s.	0.1635	n.s.	0.2675		***	0.0000	***	0.0000		***	0.0000	***	0.0000	
	**4d BV**	n.s.	0.0965	n.s.	0.1760		***	0.0000	***	0.0000		n.s.	0.0525	**	0.0014	
**GlutR Sum**	**MD**	*	0.0270				n.s.	0.4795				***	0.0000			
	**RO**	***	0.0000	**	0.0078		***	0.0000	***	0.0000		*	0.0277	***	0.0000	
	**BD**	**	0.0078	*	0.0443		***	0.0000	***	0.0000		***	0.0000	***	0.0000	
	**1hr BV**	***	0.0000	***	0.0000		***	0.0000	***	0.0000		***	0.0000	***	0.0000	
	**6hr BV**	n.s.	0.0805	n.s.	0.4991	y = 126.93-44.17*exp(-x/3.15)	n.s.	0.1701	n.s.	0.1937		n.s.	0.4714	***	0.0000	y = 55.34+64.82*exp(-x/1.20)
	**1d BV**	n.s.	0.1737	n.s.	0.4264	df=19	***	0.0000	***	0.0000		***	0.0002	***	0.0000	df=22
	**2d BV**	n.s.	0.2705	*	0.0303	R^2=0.475	***	0.0000	***	0.0000		***	0.0000	***	0.0000	R^2=0.584
	**4d BV**	n.s.	0.0671	**	0.0059	p= 0.0005	***	0.0003	**	0.0010		***	0.0000	***	0.0000	p< 0.0001
**$GABA_A$R Sum**	**MD**	**	0.0092				*	0.0157				***	0.0000			
	**RO**	**	0.0029	***	0.0000		**	0.0085	***	0.0000		n.s.	0.1604	n.s.	0.0923	
	**BD**	n.s.	0.1868	n.s.	0.2901		***	0.0000	**	0.0067		***	0.0000	***	0.0000	
	**1hr BV**	n.s.	0.4751	n.s.	0.2013		***	0.0000	***	0.0000		**	0.0031	**	0.0025	
	**6hr BV**	n.s.	0.1111	**	0.0073	y = 45.02+58.77*exp(-x/1.37)	*	0.0104	n.s.	0.4545	y = 39.14+65.54*exp(-x/1.47)	***	0.0000	***	0.0000	
	**1d BV**	***	0.0000	***	0.0000	df=19	***	0.0000	***	0.0000	df=78	*	0.0217	***	0.0000	
	**2d BV**	***	0.0000	***	0.0000	R^2=0.581	***	0.0000	***	0.0000	R^2=0.360	***	0.0000	*	0.0104	
	**4d BV**	***	0.0000	***	0.0000	p< 0.0001	***	0.0000	***	0.0000	p< 0.0001	n.s.	0.4082	n.s.	0.1277	
**$GABA_A$R Sum:GlutR Sum Index**	**MD**	**	0.0014				**	0.0088				***	0.0000			
	**RO**	n.s.	0.3428	**	0.0060		**	0.0011	n.s.	0.2762		n.s.	0.3866	***	0.0000	
	**BD**	**	0.0072	n.s.	0.0656		***	0.0000	***	0.0000		***	0.0000	***	0.0000	
	**1hr BV**	*	0.0161	n.s.	0.3428		*	0.0232	n.s.	0.3166		n.s.	0.3530	***	0.0000	
	**6hr BV**	n.s.	0.4307	*	0.0274	y = 0.73-0.76*exp(-x/3.37)	***	0.0005	n.s.	0.2605	y = 0.44-0.46*exp(-x/2.68)	***	0.0000	***	0.0000	
	**1d BV**	***	0.0000	***	0.0000	df=19	***	0.0008	***	0.0000	df=78	n.s.	0.2638	***	0.0000	
	**2d BV**	***	0.0000	***	0.0000	R^2=0.716	***	0.0005	***	0.0000	R^2=0.49	n.s.	0.2473	***	0.0001	
	**4d BV**	***	0.0002	***	0.0000	p< 0.0001	***	0.0000	***	0.0000	p< 0.0001	*	0.0177	***	0.0000	

Table 5-1. Table of p-values comparing the values for protein sums and a newly identified plasticity feature in treatment conditions against 5wk Normal animals or 5wk MD animals. p-values are presented for each cortical area (columns) and plasticity feature (rows). Cortical areas are broken up into comparisons against normal (left) and MD (right). When a curve fit was applied, the equation, degrees of freedom (df), R2 value and exact p-value are listed.

		Central					Peripheral					Monocular				
		Vs 5wk Normal		vs. 5wk MD		Curve Fit to BV Data	Vs 5wk Normal		vs. 5wk MD		Curve Fit to BV Data	Vs 5wk Normal		vs. 5wk MD		Curve Fit to BV Data
		Significance	p-value	Significance	p-value		Significance	p-value	Significance	p-value		Significance	p-value	Significance	p-value	
GABA$_A$α1:GluN2A Index	MD	***	0.0000				***	0.0000				n.s.	0.2515			
	RO	n.s.	0.3777	***	0.0000		**	0.0017	***	0.0000		n.s.	0.3474	n.s.	0.4161	
	BD	**	0.0038	n.s.	0.3288		***	0.0000	***	0.0000		***	0.0000	***	0.0000	
	1hr BV	*	0.0183	*	0.0280		***	0.0000	**	0.0046		***	0.0003	***	0.0002	
	6hr BV	**	0.0014	***	0.0000	y = 0.89-1.15*exp(-x/3.86)	***	0.0000	n.s.	0.1960	y = 0.36-0.63*exp(-x/1.87)	***	0.0000	***	0.0000	
	1d BV	*	0.0197	***	0.0000	df=22	**	0.0018	***	0.0000	df=89	***	0.0000	***	0.0000	
	2d BV	n.s.	0.1635	***	0.0000	R^2=0.732	n.s.	0.4574	***	0.0000	R^2=0.390	n.s.	0.5014	n.s.	0.4429	
	4d BV	***	0.0004	***	0.0000	p< 0.0001	*	0.0484	***	0.0000	p< 0.0001	*	0.0319	*	0.0242	
GluN2B:GluN2A Index	MD	**	0.00304				***	0				***	0.00067			
	RO	***	0	***	0		***	0	***	0		***	0	***	0	
	BD	*	0.04703	***	0.00011		***	0.00076	n.s.	0.42429		***	0	n.s.	0.33648	
	1hr BV	***	0	***	0.00078		***	0	n.s.	0.31989		***	0	***	0.00066	
	6hr BV	***	0	n.s.	0.36396	y = 0.63-0.92*exp(-x/10.98)	***	0	n.s.	0.0559	y = 0.02+-0.26*exp(-x/2.09)	n.s.	0.12763	***	0	y = -0.08-0.32*exp(-x/0.04)
	1d BV	n.s.	0.12805	*	0.03144	df=23	***	0	**	0.00142	df=77	***	0.00001	**	0.00294	df=24
	2d BV	n.s.	0.38994	*	0.02734	R^2=0.458	*	0.03331	**	0.00679	R^2=0.16	n.s.	0.10927	*	0.0435	R^2=0.240
	4d BV	*	0.01279	***	0	p=0.0002	*	0.03347	***	0	p= 0.0003	n.s.	0.29108	***	0.00027	p= 0.0111
GABA$_A$α3:GABA$_A$α1 Index	MD	***	0				***	0				n.s.	0.09922			
	RO	n.s.	0.30873	*	0.02197		**	0.00832	***	0		***	0.00081	n.s.	0.12593	
	BD	***	0.00034	n.s.	0.05618		***	0	***	0		***	0	***	0	
	1hr BV	*	0.02394	n.s.	0.08849		***	0.00036	***	0.00002		***	0	***	0	
	6hr BV	*	0.02068	*	0.03329	y = -0.29+(0.68+0.29)/(1+(x/0.09)^0.19)	***	0	***	0.00008	y = -0.16+(0.29+0.16)/(1+(x/1.76)^3.01)	***	0	***	0	
	1d BV	n.s.	0.06521	***	0	df=24	***	0	**	0.0022	df=91	**	0.00456	n.s.	0.13167	
	2d BV	***	0.00001	***	0	R^2=0.29	n.s.	0.26614	**	0.00309	R^2=0.250	n.s.	0.28274	*	0.04251	
	4d BV	n.s.	0.50054	**	0.00386	p= 0.0045	n.s.	0.11633	***	0	p< 0.0001	n.s.	0.07932	n.s.	0.21442	
GluN2B:GluA2 Index	MD	n.s.	0.1269				***	0.0000				n.s.	0.0502			
	RO	***	0.0000	***	0.0000		***	0.0000	***	0.0000		***	0.0000	***	0.0000	
	BD	n.s.	0.2035	**	0.0060		n.s.	0.3953	***	0.0001		n.s.	0.2542	**	0.0084	
	1hr BV	**	0.0041	n.s.	0.0503		***	0.0000	n.s.	0.2301		**	0.0014	n.s.	0.0559	
	6hr BV	***	0.0000	***	0.0000	y = 1.15-1.52*exp(-(x-1.61)^ 2/-5.99^2	***	0.0006	*	0.0282	y = -0.01-0.34*exp(-(x-0.83)^ 2/2.06^2	*	0.0117	***	0.0000	
	1d BV	***	0.0007	n.s.	0.0942	df=25	***	0.0000	n.s.	0.0559	df=92	n.s.	0.1674	***	0.0000	
	2d BV	***	0.0000	***	0.0000	R^2=0.231	***	0.0005	n.s.	0.0710	R^2=0.16	n.s.	0.4401	n.s.	0.0624	
	4d BV	n.s.	0.3750	*	0.0400	p=0.0112	n.s.	0.3682	***	0.0000	p< 0.0001	n.s.	0.0505	***	0.0000	
GluN2A:GluA2 Index	MD	n.s.	0.2445				n.s.	0.3563				***	0.0000	na	na	
	RO	***	0.0009	n.s.	0.0535		***	0.0000	***	0.0000		***	0.0000	***	0.0000	
	BD	*	0.0362	***	0.0004		***	0.0000	***	0.0000		***	0.0002	*	0.0125	
	1hr BV	n.s.	0.2076	*	0.0244		n.s.	0.4150	n.s.	0.4175		n.s.	0.0716	***	0.0006	
	6hr BV	n.s.	0.2357	**	0.0027		n.s.	0.0790	n.s.	0.1535		***	0.0000	*	0.0294	
	1d BV	***	0.0000	***	0.0000		*	0.0434	*	0.0127		**	0.0016	n.s.	0.1044	
	2d BV	***	0.0000	***	0.0000		n.s.	0.1986	n.s.	0.1083		n.s.	0.0887	n.s.	0.4977	
	4d BV	n.s.	0.0616	***	0.0007		n.s.	0.1366	n.s.	0.1858		n.s.	0.4026	*	0.0308	

Table 6-1. p-values comparing the each newly identified plasticity feature in treatment conditions against 5wk Normal animals and 5wk MD animals. p-values are presented for each cortical area (columns) and plasticity feature (rows). Cortical areas are broken up into comparisons against normal (left) and MD (right). When a curve fit was applied, the equation, degrees of freedom (df), R2 value and exact p-value are listed.

	Normal 1	MD 1	ST BV 1	LT BV 1	RO 2	MD 3	ST BV 3	BD 3	LT BV 4	ST BV 5	LT BV 5	LT BV 6	BD 6
**Normal 1**	1	0.97601795	0.96105886	0.98117	0.95768142	0.94818068	0.94383317	0.78413725	0.95461708	0.96535462	0.98297823	0.85942578	0.62173468
**MD 1**	0.97601795	1	0.97744298	0.99592531	0.88914347	0.95365077	0.94408703	0.76075697	0.98352939	0.9857111	0.95770586	0.89630038	0.65319854
**ST BV 1**	0.96105886	0.97744298	1	0.98000413	0.8578434	0.98158073	0.9826135	0.85467041	0.94144785	0.9899475	0.9110114	0.95290107	0.77160096
**LT BV 1**	0.98117	0.99592531	0.98000413	1	0.90193003	0.94544405	0.94037068	0.75248635	0.98758197	0.97844821	0.96541071	0.91441834	0.63534331
**RO 2**	0.95768142	0.88914347	0.8578434	0.90193003	1	0.85699952	0.85137272	0.69798422	0.8761915	0.8668415	0.9631905	0.72680318	0.47573671
**MD 3**	0.94818068	0.95365077	0.98158073	0.94544405	0.85699952	1	0.99775672	0.91971517	0.88803935	0.98380715	0.88099307	0.90030944	0.83242446
**ST BV 3**	0.94383317	0.94408703	0.9826135	0.94037068	0.85137272	0.99775672	1	0.93098098	0.87805939	0.97593087	0.87093562	0.91606814	0.84324473
**BD 3**	0.78413725	0.76075697	0.85467041	0.75248635	0.69798422	0.91971517	0.93098098	1	0.64415169	0.84073526	0.66177201	0.79637235	0.94438398
**LT BV 4**	0.95461708	0.98352939	0.94144785	0.98758197	0.8761915	0.88803935	0.87805939	0.64415169	1	0.94535345	0.96449423	0.87660664	0.52623206
**ST BV 5**	0.96535462	0.9857111	0.9899475	0.97844821	0.8668415	0.98380715	0.97593087	0.84073526	0.94535345	1	0.92400086	0.90578365	0.75550348
**LT BV 5**	0.98297823	0.95770586	0.9110114	0.96541071	0.9631905	0.88099307	0.87093562	0.66177201	0.96449423	0.92400086	1	0.79577094	0.47606361
**LT BV 6**	0.85942578	0.89630038	0.95290107	0.91441834	0.72680318	0.90030944	0.91606814	0.79637235	0.87660664	0.90578365	0.79577094	1	0.76469463
**BD 6**	0.62173468	0.65319854	0.77160096	0.63534331	0.47573671	0.83242446	0.84324473	0.94438398	0.52623206	0.75550348	0.47606361	0.76469463	1

Table 8-1. Pearson's R values comparing the strength of association between each treatment subcluster. The correlation between each sub-cluster was measured and the observed R values are presented in a matrix. This matrix was reordered in Figure 8 to position high R values nearest one another.

	Normal 1	MD 1	ST BV 1	LT BV 1	RO 2	MD 3	ST BV 3	BD 3	LT BV 4	ST BV 5	LT BV 5	LT BV 6	BD 6
**Normal 1**	NA	0.00003	0.00014	0.00002	0.00018	0.00033	0.00042	0.02125	0.00023	0.0001	0.00001	0.00623	0.09983
**MD 1**	0.00003	NA	0.00003	0	0.00313	0.00024	0.00042	0.02839	0.00001	0.00001	0.00018	0.00258	0.07903
**ST BV 1**	0.00014	0.00003	NA	0.00002	0.00644	0.00002	0.00001	0.00686	0.00048	0	0.00165	0.00025	0.02492
**LT BV 1**	0.00002	0	0.00002	NA	0.00219	0.00039	0.00051	0.03122	0	0.00002	0.0001	0.00147	0.09049
**RO 2**	0.00018	0.00313	0.00644	0.00219	NA	0.00655	0.00732	0.05421	0.00431	0.00533	0.00012	0.0411	0.23344
**MD 3**	0.00033	0.00024	0.00002	0.00039	0.00655	NA	0	0.00122	0.00322	0.00001	0.00385	0.0023	0.01034
**ST BV 3**	0.00042	0.00042	0.00001	0.00051	0.00732	0	NA	0.00078	0.00413	0.00003	0.00487	0.00139	0.00853
**BD 3**	0.02125	0.02839	0.00686	0.03122	0.05421	0.00122	0.00078	NA	0.08473	0.00893	0.07385	0.01802	0.00041
**LT BV 4**	0.00023	0.00001	0.00048	0	0.00431	0.00322	0.00413	0.08473	NA	0.00039	0.00011	0.00427	0.18034
**ST BV 5**	0.0001	0.00001	0	0.00002	0.00533	0.00001	0.00003	0.00893	0.00039	NA	0.00104	0.00195	0.03017
**LT BV 5**	0.00001	0.00018	0.00165	0.0001	0.00012	0.00385	0.00487	0.07385	0.00011	0.00104	NA	0.01817	0.23308
**LT BV 6**	0.00623	0.00258	0.00025	0.00147	0.0411	0.0023	0.00139	0.01802	0.00427	0.00195	0.01817	NA	0.02709
**BD 6**	0.09983	0.07903	0.02492	0.09049	0.23344	0.01034	0.00853	0.00041	0.18034	0.03017	0.23308	0.02709	NA

Table 8-2. Bonferroni corrected p-values between each treatment subcluster. The Bonferroni corrected p-values were used to identify the most significant correlations between proteins. P-values less than the Bonferroni corrected level (0.0006) are coloured red to simplify identification of significant correlations.

	Comparison	MD 1	LT BV 1	ST BV 1	RO 2	MD 3	ST BV 3	BD 3	LT BV 4	ST BV 5	LT BV 5	LT BV 6	BD 6
**All Protein Sum**	pvalue	0	0	0	0	0	0	0	0	0.01366	0.00003	0	0.00062
	Asterisk	***	***	***	***	***	***	***	***	*	***	***	***
	color	red	red	red	red	red	red	red	red	blue	blue	blue	blue
**GlutR Sum**	pvalue	0	0	0	0	0	0	0.00006	0	0.00001	0	0.03855	0.00004
	Asterisk	***	***	***	***	***	***	***	***	***	***	*	***
	color	red	red	red	red	red	red	red	red	red	red	red	blue
**$GABA_A$R Sum**	pvalue	0.00281	0	0	0	0	0	0	0	0	0	0	0.39037
	Asterisk	**	***	***	***	***	***	***	***	***	***	***	n.s.
	color	blue	blue	blue	blue	red	red	red	blue	blue	blue	blue	white
**GlutR:$GABA_A$R Sum**	pvalue	0	0	0	0	0	0	0	0	0	0	0	0.0001
	Asterisk	***	***	***	***	***	***	***	***	***	***	***	***
	color	red	red	red	red	red	red	blue	red	red	red	red	blue
**$GABA_A$α1:GluN2A**	pvalue	0	0	0	0	0	0	0	0.27593	0	0.41887	0	0
	Asterisk	***	***	***	***	***	***	***	n.s.	***	n.s.	***	***
	color	red	red	red	red	red	red	red	white	red	white	red	red
**GluN2B:GluN2A**	pvalue	0.00206	0.02562	0.17913	0	0.00009	0.2111	0.30192	0.31158	0.00968	0	0	0.00003
	Asterisk	**	*	n.s.	***	***	n.s.	n.s.	n.s.	**	***	***	***
	color	blue	red	white	red	blue	white	white	white	blue	red	red	blue
**$GABA_A$α1:$GABA_A$α3**	pvalue	0.00017	0.00009	0	0.0205	0	0	0	0.03374	0.02511	0	0	0
	Asterisk	***	***	***	*	***	***	***	*	*	***	***	***
	color	red	red	red	red	red	red	red	red	blue	blue	red	red
**GluN2B:GluA2**	pvalue	0	0	0.00011	0	0	0	0	0.01204	0.00196	0	0	0
	Asterisk	***	***	***	***	***	***	***	*	**	***	***	***
	color	red	red	red	red	red	red	red	red	red	red	red	red
**GluN2A:GluA2**	pvalue	0	0	0	0	0	0	0	0	0	0.00001	0.01706	0
	Asterisk	***	***	***	***	***	***	***	***	***	***	*	***
	color	red	red	red	red	red	red	red	red	red	red	red	red

Table 10-1. p-values for each identified plasticity feature within subclusters compared against the Normal animals from cluster 1.p-values are presented for the Pearson's R correlations between each plasticity phenotype and the Normal subcluster. The corresponding significance level is indicated by the text colour red if the value was significantly above the normal subcluster, and blue if the value was significantly below, and white if not significantly different.

		Central					Peripheral					Monocular				
		Vs 5wk Normal		vs. 5wk MD		Curve Fit to BV Data	Vs 5wk Normal		vs. 5wk MD		Curve Fit to BV Data	Vs 5wk Normal		vs. 5wk MD		Curve Fit to BV Data
		Significance	p-value	Significance	p-value		Significance	p-value	Significance	p-value		Significance	p-value	Significance	p-value	
**NMDAR Predicted Decay Kinetics**	**MD**	**	0.00312				***	0				***	0.0007			
	**RO**	***	0	***	0		***	0	***	0		***	0	***	0	
	**BD**	n.s.	0.0836	***	0		***	0	n.s.	0.22522		***	0	n.s.	0.33676	
	**1hr BV**	***	0	***	0.00082		***	0	n.s.	0.42141		***	0	***	0.00053	
	**6hr BV**	***	0	n.s.	0.36066	y = -126.4+257.7*exp(-x/12.41)	***	0	*	0.01421	y = 63.9+53.77*exp(-x/1.76)	*	0.03776	***	0	
	**1d BV**	n.s.	0.12732	*	0.03145	df=23	***	0	***	0.00002	df=87	***	0	**	0.00303	
	**2d BV**	n.s.	0.29523	*	0.02565	R^2=0.444	*	0.01772	***	0.00063	R^2=0.153	*	0.0441	*	0.04309	
	**4d BV**	**	0.00108	***	0	p= 0.0003	n.s.	0.07203	***	0	p= 0.0002	n.s.	0.17444	***	0	
**$GABA_AR$ Predicted Decay Kinetics**	**MD**	***	0				***	0.00035				n.s.	0.37952			
	**RO**	n.s.	0.30147	n.s.	0.08113		n.s.	0.15855	n.s.	0.0877		***	0	***	0	
	**BD**	***	0	n.s.	0.05567		***	0	***	0		***	0	***	0	
	**1hr BV**	n.s.	0.22184	n.s.	0.09753		n.s.	0.05472	n.s.	0.05363		***	0	***	0	
	**6hr BV**	**	0.00871	*	0.04659	y = 448.9+(48.16-448.9)/(1+(x/6.49)^7.85)	***	0	***	0	y = 63.99+(48.05-63.99)/(1+(x/1.84)^16.59)	***	0	***	0	
	**1d BV**	*	0.02853	***	0.00001	df=24	***	0	n.s.	0.09948	df=91	***	0	***	0	
	**2d BV**	***	0	***	0.00001	R^2=0.39	*	0.02597	***	0.00013	R^2=0.327	n.s.	0.27708	n.s.	0.20512	
	**4d BV**	n.s.	0.10545	**	0.00805	p=0.0006	**	0.00938	***	0.00003	p< 0.0001	n.s.	0.10039	n.s.	0.19565	

Table 11-1. p-values for predicted kinetics among treatment conditions compared against the 5 week Normal and 5 week MD animals. p-values are presented for each cortical area (columns) and for the predicted kinetics of each receptor type(rows). Cortical areas are broken up into comparisons against normal (left) and MD (right). When a curve fit was applied, the equation, degrees of freedom (df), R2 value and exact p-value are listed.
